# Prenatal effects by exposing to amoxicillin on dental enamel in Wistar rats

**DOI:** 10.4317/medoral.18807

**Published:** 2013-10-13

**Authors:** Beatriz Gottberg, Jeanily Berné, Belkis Quiñónez, Eduvigis Solórzano

**Affiliations:** 1 DDS, University of Los Andes, Mérida-Venezuela; 2 Professor of Pharmacology, Department of Pathobiology, Faculty of Dentistry, Universidad de Los Andes, Mérida-Venezuela. Pharmacology and Toxicology, Faculty of Medicine Medical. University of Los Andes, Mérida-Venezuela; 3 PhD, Professor of Histology, Research Group Biopathological/Lab integrated by Cellular and Molecular Biology, Department of Pathobiology, Faculty of Dentistry, University of Los Andes, Mérida-Venezuela

## Abstract

Amoxicillin is an antibiotic widely prescribed; its most frequent side effects are gastrointestinal disorders and hypersensitivity reactions. Over the last 10 years studies have been published which suggest that amoxicillin may cause dental alterations similar to dental fluorosis. Never the less, the results are not conclusive, this is why it was planned the need to make controlled studies on test animals. Objectives: The purpose of this study was to determine the effect produced by amoxicillin prenatal administration on dental enamel in Wistar rats. Study Design: 12 pregnant adult rats were used distributed into five different groups: witness control (n=2) didn’t get any treatment; negative control (n=2) they were prescribed with saline solution; positive control (n=3) they were prescribed with tetracycline 130 mg/kg, and two groups (n=3 and n=2) treated with amoxicillin doses of 50 and 100 mg/kg respectively. The treatments were daily administered by mouth, from the 6th gestation day to the end of gestation. Twenty five days after they were born, the offspring were sacrificed with a sodium pentobarbital overdose, the mandible was dissected and the first lower molars were gotten. The samples were fixed in 10% formaldehyde solution and clinically and histologically observed to determine any enamel disorders. Results: hypomineralization was observed in every single sample of the tetracyclic and amoxicillin treated group 100 mg/kg, meanwhile only 50% from the group administered with 50 mg/kg amoxicillin showed this histological disorder. Conclusions: the side effect caused by amoxicillin on dental enamel was doses dependent.

** Key words:**Amoxicillin, dental enamel, hypomineralization, Wistar rats.

## Introduction

Amoxicillin is semisynthetic wide spectrum penicillin, with bactericide activity against gram-positive and gram-negative bacteria. Most of all, it’s prescribed as first choice antibiotic for respirational, gastrointestinal, genital, cutaneous, and neurological infection treatment (1). As well as, for the dentists area is usually prescribed against microorganisms associated with dental-alveolar abscess, soft tissue, maxillary and oral sinus communications infections (2); it is also the first choice medication to prevent infectious endocarditis (3).

The most frequent side effects triggered by this antibiotic are hypersensitivity reactions and gastrointestinal disorders (1). Fur-thermore, these last years, clinical cases and epidemiology studies have been published (4-9) which suggest that amoxicillin may cause dental pathologies like dental fluorosis. The first cause for this disorder is an excess fluoride ions intake during dental formation period, histologically characterized by enamel hypomineralization and the clinical sign which identifies it, a subtle white mottled mixed in a dark brown hole (5). Even though epidemiology study authors determine that the epidemiology of hypomineralization is not clear yet, they suggest that kids who have had therapies with amoxicillin are more likely to develop dental disorders, reason why there should be more research on these dental lesions using antibiotics (9).

On lab animals, has been studied this possible amoxicillin side effect. In this sense, Laisi et al. (8) were observed an increase of enamel thickness on mice teeth on line E18 flooded into amoxicillin solution, not affecting teeth; this finding was attributed by the authors by a temporal normal sequence alteration from the enamel development stages. However, the results from a recent published study shows that of a single dose prescription of amoxicillin in Wistar rats affect dental mineralization on incisive teeth but not on the enamel (10).

Amoxicillin’s widespread use in pregnant women as well as in children, besides the lack of conclusive evidence on its effect on the development of dental enamel, suggest the need to establish if there’s a link with the prescription of this medication and dental disorders.

Considering that live studies done in lab animals allow a rigorous research on pharmacological side effects; and that the odontogenesis process and dental histological structure from some test animals are similar to humans, it was proposed as an objective for this study to determine if the prescription of amoxicillin during prenatal period cause disorders on dental enamel on Wistar rats.

## Material and Methods

Thirty Wistar rats were used in the present study, coming from the Bioterio from University of Los Andes (BIOULA), Mérida-Venezuela, female, adult, nulliparous, weighing approximately 200g each, the rats were kept in cages at room temperature and were given ad libitum access to food and water.

The rats on proestrus or oestrus stage (diagnosed by vaginal smear), were paired adult males from the same line, in 1:1 proportion. Twenty four hours later the rat vagina were inspected and the presence of a mucous vaginal tampon was considered as a mating indicator, registered as the gestation first day (11).

On the gestation third day, the animals were taken to Department of Pharmacology and Toxicology from Faculty of Medicine (ULA), to allow adaptation to new environmental conditions before starting treatments, keeping feeding and water ad libitum. On the sixth day of gestation, rats were assigned at random to the five study groups ( Table 1) and placed in separate metal cages labeled according the group study they belonged to.

**Table 1:**
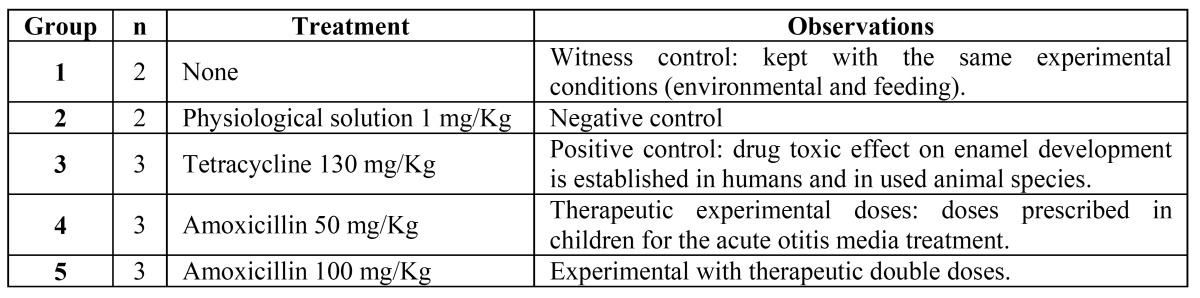
Study groups distribution.

Treatments were administered in a daily dose, by mouth, with a metal esophageal catheter from the sixth day to the end of gestation. Two hours before applying treatments, food was taken away and they were weighted every other day to calculate corresponding single doses. At the end of the gestation process the birthdate was registered for the offspring and were kept together with the mothers till weaning.

To obtain the study molars, day twenty four after birth, animals were sacrificed by an intraperitoneal shot with a sodium pentobarbital overdose in 6 mg/ml solution. The mandibles were dissected and fixed in 10% formaldehyde solution, in individually identified vessels by the corresponding group. After 48 hours of fixation, the mandibles were dissected in the middle of the anterior segment and the first lower molars were isolated (right or left), cutting by mesial and distal, with a low speed micromotor and soflex discs.

For microscopic observation, samples were set by the wearing down method using the same hemi mandibles and a micromotor with soflex discs, the vestibular and lingual surfaces were worn down in a mesiodistal way, to obtain layers of .5 µm thicknesses which were submerged in xylol, to clean and make easier for further microscopic observation. The samples, in groups of 6, were placed on clean and dry trays, a few drops of Martex® were added, as a mounting mean and were covered by lamina allowing it to dry for 24 hours, then the observation was made with a LEICA®, model DNR-HC, trinocular microscope. All analyses were performed by the same calibrated examiner, who did not know to which group the sections belonged. Histologic and clinical changes were registered in an exclusively designed index card for this study. For statistical analysis, the independent variable was treatment, whereas the dependent variable was enamel alteration.

Bioethical Aspects: for this research the established regulation for animals lab use for teaching and research purposes, was applied (12). For this reason the amount of animals selected was the lowest necessary to obtain accurate results with all the basic handle and experimentation cares throughout all the investigational stages on living animals.

## Results

Clinical findings: all molars were clinically observed (81 first lower molars right or left), only one molar from each animal from the brood. In every single case the dental anatomy characteristics for this kind of animals were observed without any variation, with 3 important picks, from a functional point of view, located in vestibular, highest diameter vestibule-lingual in distal and the lowest diameter vestibule-lingual in mesial, with 4 roots, 1 mesial, another broad distal and another 2 thin and narrow located in the middle, one towards lingual and the other towards vestibular. No agenesis was observed or any change on dental development in the brood.

Animals were wean after 21 days and sacrificed on the 25th day, this is why on most of these cases there were observed food leftovers in the molar occlusal side grooves, these leftovers were removed with a medium size bristles brush, fully cleaning the enamel surface. Nevertheless, the group treated with tetracycline one of the samples exposed enamel pigmentation.

Histological findings: samples were randomly selected with a total of 10 molars per group. During the wearing down technique process, some of the teeth fractured or were missing due to their size, leaving only 6 samples in good conditions for each group, with an average thickness of 10 µm, for a total of 30 samples.

**Figure 1:**
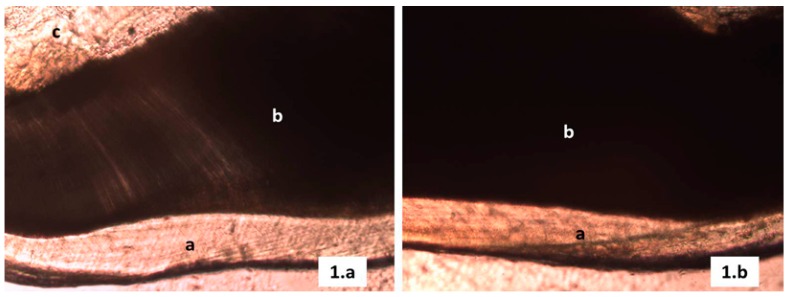
a) Photomicrography mesio-distal cut, witness group tooth. a Enamel, b Dentine, c Pulp chamber (magnification gives 10X, wearing down technique). b) Photomicrography mesio-distal cut tooth from the negative control group. a enamel, b dentine, c pulp chamber (original magnification 10X, wearing down technique).

**Figure 2:**
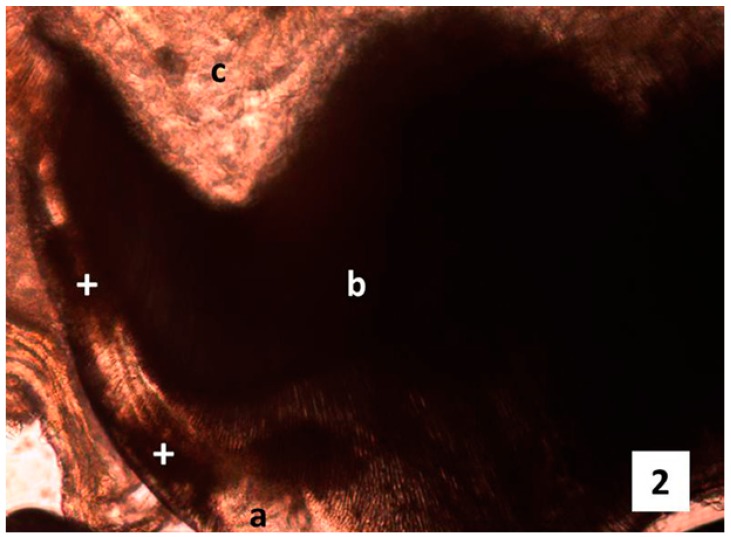
Photomicrography mesio-distal cut, Positive control group tooth tetracycline therapeutic doses. a Enamel, b Dentine, c Pulp chamber + enamel hypomineralization areas (original magnification 10X, wearing down technique).

The structural analysis thru the optical microscope revealed for all the samples in groups 1 and 2 (witness control and negative control respectively), comparable lines with less mineral contents located from the amelodentinal junction to the open surface which belong to the incremental lines or striae of Retzius (Fig. 1). On the other hand, for the samples on the positive control group (treated with tetracycline 130 mg/Kg), several shapes of shady areas were observed over most of the enamel, those areas related to organic matrix inadequate mineralization during enamel development (Fig. 2).

**Figure 3:**
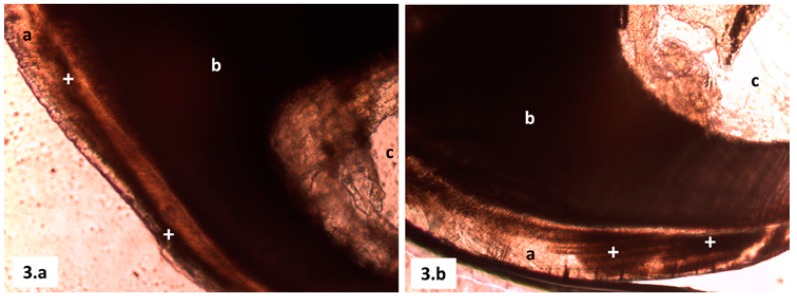
a) Photomicrography mesio-distal cut, Experimental group tooth amoxicillin therapeutic doses. a Enamel, b Dentine, c Pulp chamber + enamel hypomineralization areas (magnification gives 10X, wearing down technique). b) Photomicrography mesio-distal cut, experimental group tooth amoxicillin therapeutic double doses. a Enamel, b Dentine, c Pulp chamber + enamel Hypomineralization areas (original magnification 10X, wearing down technique).

For the experimental groups 4 and 5 (treated with amoxicillin 50 mg/Kg and with amoxicillin 100 mg/Kg, respectively) it was observed on the enamel areas with different color shades from light brown to dark brown, representing areas of hypomineralization (Fig. 3), even in one of the samples from group 4 it was observed a very dark stripe along the enamel as a striae of Retzius, very dusky, which usually results from systematic disorder cases (Fig. 3). Distribution of enamel alteration per group is evident in  Table 2.

**Table 2:**
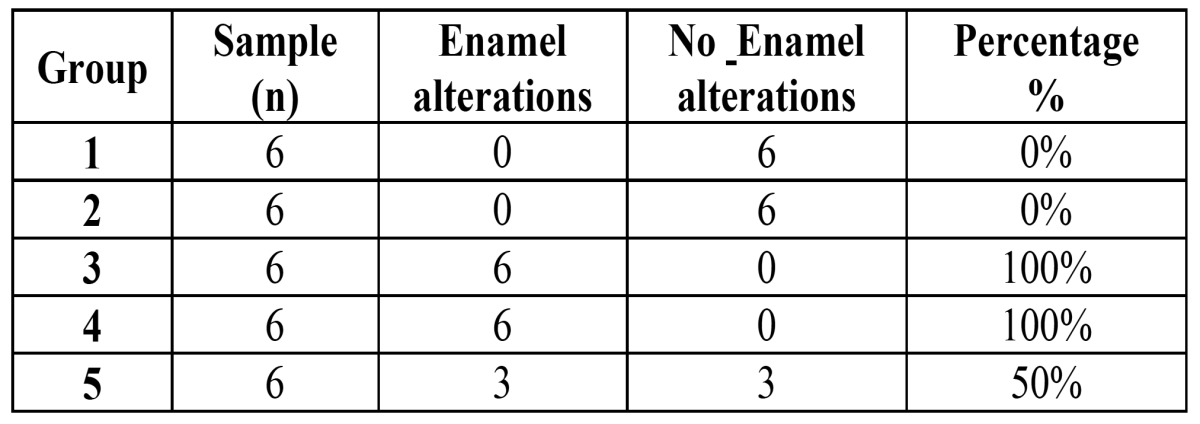
Enamel alterations distribution per group.

It’s important to highlight that the enamel’s thickness was quite similar for all the groups, even though, the wearing down technique and how fragile the samples from the groups treated with tetracycline 130 mg/Kg and amoxicillin 100 mg/Kg, there was loss of adamantine tissue continuity.

## Discussion

In order to corroborate other research (4-10) suggesting that amoxicillin causes alterations in dental enamel development, this preclinical study was conducted using a positive control group which was given tetracycline.

According to the chronology of tooth eruption in rats on day 25 after birth all animals presented the first and second molars (13). This finding suggests that prenatal amoxicillin and tetracycline prescription, didn’t affect the eruption neither the development of molar dental germs (14).

The presence of light pigmentations on occlusal grooves in one of the molars from the group exposed to tetracycline (130 mg/kg), coincides with reports of several authors (15,16), who refer that dental alterations caused by these drugs are optically visible. Likewise, all molars exposed to tetracycline showed hypomineralized enamel, relating to several published studies (15,17), where it has been widely made known that tetracycline cause structural and functional changes in ameloblasts and in immature enamel; therefore, its use as positive control is valid to assess alterations in dental enamel caused by medications and other toxic substances in lab animals (15,17).

Despite, alteration evidences were not clinically observed on the molar dental enamel rat offspring from mothers which received amoxicillin in therapeutic doses and double doses; in the histological analysis were observed enamel lesions which were identified as hypomineralization areas, also called opacities, defined as qualitative flaws from dental tissues, identified as an abnormality in the enamel’s translucently, characterized by white, tan, yellow, or brown areas and of smooth surface (18).

The presence of hypomineralization areas on the rat offspring molars exposed to amoxicillin during prenatal period, relates with results from several clinical researches, where they report that children who have received amoxicillin are at more risk to develop future dental problems (4-9). Most of these authors describe the findings of hypomineralization, while others refer to dental fluorosis (5,6).

As well, the results of this study coincide with those observed in an in vitro study (8). In the study, amoxicillin produced hipomineralization on tooth enamel mice E18 this effect was associated with an altered pattern of amelogenesis, due to the modification of the normal temporal sequence of dental enamel development. Based on this antecedent, we believe that is possible that in this current research, there may be an alteration in the amelogenesis temporal patter.

Hypomineralized enamel was observed in all group samples treated with amoxicillin at a 100 mg/Kg doses, while only 50% of the samples from the group which received 50 mg/Kg doses presented these changes, having lesion differences, given that the group with therapeutic doses, hypomineralization was observed in a striped shape or fringe located on the enamel’s verge, while the group with double therapeutic doses, besides the stripes, there were defined hypomineralization areas, extended throughout the enamel’s thickness. These differences show that the effect caused by amoxicillin on the dental enamel was doses dependent. Similar results have been published in some in vitro studies (8,19). Amoxicillin incorporation in developing tooth enamel can be favored by its liposolubility (superior to other penicillin), for the wide range of blood vessels found in the intermediate layer in the molar intercusp regions and by the little affinity this medication has because of plasmatic proteins (1,17).

The mechanism by which some antibiotics affecting dental enamel has been attributed to altered protein synthesis that these drugs produce (20). Recently, Sahlberg et al. (19) demonstrated that amoxicillin decreases MMP20 expression, a metalloproteinase which has an important role in the degradation and removal of enamel proteins. However, to clarify what is the hypomineralization mechanism further research is necessary, with methodological designs to evaluating the cellular and molecular actions of amoxicillin.

In contrast to previous study also performed in vivo in Wistar rats (21), in this investigation we did not find changes in dentine. This may be due to differences in dosage, routes of administration and duration of the treatments applied. Also, the divergence in results could be attributed to the study cited evaluated incisors.

Even with its limitations, this study demonstrates the importance of reassessing the safety of administration of amoxicillin during pregnancy and early childhood. Similarly, the results suggest the need to design future research, including evaluation of hypomineralization cause in dental enamel with amoxicillin use.

## Conclusion

The observed hypomineralization in the enamel of rats Wistar toffspring confirms the research hypothesis and suggests that the effect caused by the amoxicillin prenatal administration is doses dependent.
